# Association Between Dietary Habits and Depressive Symptoms in University Students: A Cross-Sectional Study Using the Japanese Version of the Quick Inventory of Depressive Symptomatology (QIDS-J)

**DOI:** 10.7759/cureus.87140

**Published:** 2025-07-01

**Authors:** Yuusuke Harada, Misako Homma, Michiko Miyakawa

**Affiliations:** 1 Graduate School of Public Policy, Hosei University, Tokyo, JPN; 2 Graduate School of Humanities and Social Sciences, Hiroshima University, Hiroshima, JPN

**Keywords:** carbohydrates, depressive symptoms, dietary habits, qids-j, university students

## Abstract

Objective

Amid growing concerns about mental health among young people in Japan, particularly the rising suicide rate linked to depression, this study aimed to cross-sectionally investigate the association between dietary habits and depressive symptoms in university students, from the perspective of nutritional psychiatry.

Methods

A self-administered questionnaire survey was conducted using Google Forms (Google, Inc., Mountain View, CA, USA), with 451 students at Hosei University in 2022. The survey consisted of an original questionnaire on dietary habits and the Japanese version of the Quick Inventory of Depressive Symptomatology (QIDS-J). Based on the QIDS-J scores, participants were divided into two groups (with or without depressive symptoms), using a cutoff score of 6. Logistic regression analysis was used to identify associated dietary factors.

Results

Of the valid respondents, 46.3% exhibited mild or more severe depressive symptoms (QIDS-J ≥ 6). Logistic regression analysis revealed that factors significantly associated with depressive symptoms were the consumption of high-carbohydrate meals, skipping breakfast, having a nutritionally imbalanced diet, self-awareness of irregular eating habits, and a desire to diet. The consumption of high-carbohydrate meals was suggested to have the strongest association. Furthermore, the association regarding the intake of soft drinks was reversed between athletic dormitory students and general students, indicating that lifestyle differences acted as a confounding factor.

Conclusion

This study suggests that unhealthy dietary habits among university students are potentially associated with the severity of depressive symptoms. In particular, a carbohydrate-centered diet is presumed to have a significant impact on mental health. Future longitudinal studies are necessary to determine the causal relationships in greater detail.

## Introduction

In recent years, suicide among young people has been on the rise in Japan. Suicide is the leading cause of death for individuals aged 10-39, accounting for over 50% of all deaths in the 15-29 age group, in particular [1]. This demographic primarily consists of high school and university students, with mental disorders - mainly depression - reported as the most common underlying factor [2]. While various factors contribute to mental health, the brain, which is the foundation of mental well-being and is affected by psychiatric disorders, relies on nutrients such as lipids, amino acids, vitamins, and minerals. This understanding led to the establishment of the International Society for Nutritional Psychiatry Research in 2013, stimulating research on the link between diet and depression [3]. The World Health Organization (WHO) also recommends nutritional improvements to reduce the potential risk of mental disorders [4].

Meanwhile, university students are known to have irregular dietary habits, such as skipping breakfast, excessive carbohydrate intake, and high consumption of soft drinks. Previous studies have reported that higher interpersonal stress is associated with less attention to nutritional balance [5], an increase in the rate of skipping breakfast [6], a link between nutritional imbalance and impulsivity [7], and an association between sleep/eating habits and mental health [8]. However, the relationship between dietary habits and depressive symptoms has not been fully elucidated.

Therefore, this study aimed to conduct a cross-sectional investigation of the association between depressive symptoms and dietary factors among university students, using the Quick Inventory of Depressive Symptomatology-Japanese version (QIDS-J), a self-report scale for depressive symptoms, and an original dietary questionnaire. It is important to note that, due to the cross-sectional design of this study, the investigation focuses on identifying associations rather than establishing causal relationships.

## Materials and methods

Subjects and procedure

The subjects of this study were 455 undergraduate students enrolled in the "Health and Public Hygiene" course at Hosei University in the 2022 academic year. This study was approved by the Ethics Review Committee of Hosei University (approval no. N2022-120012). The required sample size was calculated using G*Power software (Heinrich-Heine-Universität Düsseldorf, Düsseldorf, Germany), assuming a significance level (α error) of 0.05, power of 0.80, and an effect size of 1.5. The resulting minimum required sample size was 354, which the number of participants in this study exceeded.

Data collection was conducted from November to December 2022. The survey was a self-administered questionnaire distributed online using Google Forms (Google, Inc., Mountain View, CA, USA). Prior to their participation, subjects were provided with a written explanation of the study's purpose, the voluntary nature of their participation, and the protection of personal information, after which their electronic consent was obtained.

Measures

Questionnaire on Dietary Habits and Related Factors

An original 31-item questionnaire was used to measure dietary habits and related factors (see Appendix). The main items measured were as follows: basic attributes (gender, age, etc.), weekly intake of soft drinks (in 500 mL units), frequency of breakfast consumption, frequency of convenience store use, frequency of high-carbohydrate meals, frequency of nutritionally balanced meals, self-awareness of irregular eating habits compared to before entering university, presence of a desire to diet, and the frequency of use and types of dietary supplements.

To supplement the quantitative data, participants were asked to provide a free-text response of at least 100 Japanese characters on why they believe nutrition is, or is not, related to mental health.

Depressive Symptoms

Depressive symptoms were assessed using the QIDS-J. The QIDS-J is a 16-item self-report scale that can evaluate the severity of depression and corresponds to the diagnostic criteria for Major Depressive Disorder in the DSM-IV. The score is calculated from the sum of nine items (the highest-scoring item from each of the domains of sleep, appetite/weight, and psychomotor status, plus six other items), with a total score ranging from 0 to 27. The severity is classified as follows: 0-5 (normal), 6-10 (mild), 11-15 (moderate), 16-20 (severe), and 21-27 (very severe) [9]. The reliability and validity of the QIDS-J have been verified by Fujisawa et al. [10]. It should be noted that the QIDS-J, the scale used in this study, is in the public domain and available for use by anyone without permission.

Analysis strategy

Statistical analysis was performed using IBM SPSS Statistics for Windows, Version 29 (released 2022; IBM Corp., Armonk, NY, USA), and the level of statistical significance was set at p < 0.05.

As the primary analysis, logistic regression (forced entry method) was conducted to identify dietary factors affecting the presence of depressive symptoms. For the analysis, the dependent variable was dichotomized based on the total QIDS-J score into two groups: "no depressive tendency" (score ≤ 5) and "depressive tendency" (score ≥ 6), in accordance with previous research [11]. The explanatory variables were the items from the questionnaire on dietary habits and related factors. Items measured on a Likert scale were treated as continuous variables in the analysis.

Furthermore, a preliminary analysis revealed a significant difference in soft drink consumption between dormitory and non-dormitory students (p < 0.05). Therefore, referencing the study by Narita et al. [12], a sub-analysis was conducted using the same procedure on a sample of 400 participants, after excluding dormitory residents (n = 51).

Finally, to supplement the quantitative analysis, a qualitative examination was conducted on the characteristics of individual participants whose QIDS-J scores were classified as "very severe."

## Results

The final analysis included 451 participants after excluding responses with missing values or incorrect entries. The sample consisted of 183 females (40.6%) and 268 males (59.4%). The mean age of the participants was 21.1 years (SD = 4.5). The mean QIDS-J score for the entire sample was 5.8 (SD = 3.9). For dormitory students (primarily student-athletes), the mean score was 4.5 (SD = 2.6), while for other students, it was 6.0 (SD = 4.0) (Table 1).

**Table 1:** Participant Characteristics and Mean QIDS-J Scores by Group *The difference between the two groups was statistically significant, t(60.03) = 4.13, p < 0.001. Values in parentheses are rounded to 3 decimal places. QIDS-J, Quick Inventory of Depressive Symptomatology-Japanese Version

Characteristic/Item		Dormitory Residents (n = 51)	Other Students (n = 400)	Overall (n = 451)
Age (years; mean ± standard deviation)		20.3 ± 0.7	21.3 ± 4.8	21.1 ± 4.5
Gender (n(%))				
Male		49 (96.1%)	219 (54.8%)	268 (59.4%)
Female		2 (3.9%)	181 (45.3%)	183 (40.6%)
Height (cm; mean ± standard deviation)		174.0 ± 6.1	166.7 ± 9.2	167.6 ± 9.2
Weight (kg; mean ± standard deviation)		78.1 ± 13.2	58.2 ± 11.4	60.5 ± 13.2
BMI (mean ± standard deviation)		25.8 ± 3.7	20.8 ± 2.7	21.4 ± 3.3
Sugary drink consumption (bottles/week; mean ± standard deviation)		4.7 ± 3.8	2.4 ± 3.3*	2.7 ± 3.4
Breakfast consumption frequency (persons; n(%))				
0 times		2 (3.9%)	49 (12.3%)	51 (11.3%)
1-2 times (rarely eat)		12 (23.5%)	95 (23.8%)	107 (23.7%)
3-4 times (eat about half the week)		7 (13.7%)	83 (20.8%)	90 (20.0%)
5-6 times (almost always eat)		19 (37.3%)	66 (16.5%)	85 (18.8%)
7 times (always eat)		11 (21.6%)	107 (26.8%)	118 (26.2%)
Convenience store use frequency (persons; n(%))				
0 days (never use)		6 (11.8%)	63 (15.8%)	69 (15.3%)
1-2 days (rarely use)		29 (56.9%)	218 (54.5%)	247 (54.8%)
3-4 days (use about half the week)		13 (25.5%)	90 (22.5%)	103 (22.8%)
5-6 days (almost always use)		1 (2.0%)	21 (5.3%)	22 (4.9%)
7 days (always use)		2 (3.9%)	8 (2.0%)	10 (2.2%)
Meals consisting only of high-carbohydrate items (persons; n(%))				
Almost every day		1 (2.0%)	35 (8.8%)	36 (8.0%)
More than half the week		15 (29.4%)	86 (21.5%)	101 (22.4%)
About half the week		8 (15.7%)	108 (27.0%)	116 (25.7%)
Less than half the week		23 (45.1%)	142 (35.5%)	165 (36.6%)
Never		4 (7.8%)	29 (7.3%)	33 (7.3%)
Consumption of nutritionally balanced meals (persons; n(%))				
Almost every day		24 (47.1%)	76 (19.0%)	100 (22.2%)
More than half the week		11 (21.6%)	88 (22.0%)	99 (22.0%)
About half the week		5 (9.8%)	117 (29.3%)	122 (27.1%)
Less than half the week		9 (17.6%)	93 (23.3%)	102 (22.6%)
Never		2 (3.9%)	26 (6.5%)	28 (6.2%)
Perceived dietary disruption (persons; n(%))				
Agree		30 (58.9%)	220 (55.0%)	250 (55.4%)
Somewhat agree		11 (21.6%)	96 (24.0%)	107 (23.7%)
Somewhat disagree		6 (11.8%)	48 (12.0%)	54 (12.0%)
Disagree		4 (7.8%)	36 (9.0%)	40 (8.9%)
Desire to diet (persons; n(%))				
Agree		10 (19.6%)	140 (35.0%)	150 (33.3%)
Somewhat agree		8 (15.7%)	66 (16.5%)	74 (16.4)
Somewhat disagree		12 (23.5%)	50 (12.5%)	62 (13.7%)
Disagree		21 (41.2%)	144 (36.0%)	165 (36.6%)
Health auxiliary foods (persons; n(%))				
Almost every day		22 (43.1%)	84 (21.0%)	106 (23.5%)
More than half the week		4 (7.8%)	13 (3.3%)	17 (3.8%)
About half the week		2 (3.9%)	19 (4.8%)	21 (4.7%)
Less than half the week		5 (9.8%)	63 (15.8%)	68 (15.1%)
Never		18 (35.3%)	221 (55.3%)	239 (53.0%)
QIDS-J score (points; mean ± standard deviation)		4.5 ± 2.6	6.0 ± 4.0	5.8 ± 3.9

The main analysis identified six factors associated with QIDS-J scores: high consumption of soft drinks, skipping breakfast, consumption of high-carbohydrate meals, non-consumption of nutritionally balanced meals, self-awareness of irregular eating habits, and a desire to diet (Tables 2-3).

**Table 2:** Logistic Regression Analysis Predicting QIDS-J Score ≥ 6 Among All Participants (n = 451) r, Correlation Coefficient; β, Standardized Partial Regression Coefficient; QIDS-J, Quick Inventory of Depressive Symptomatology-Japanese Version

Independent Variable	r	β	Odds Ratio	95% CI (Lower Bound)	95% CI (Upper Bound)	p-value
Sugary drinks	0.13	-0.22	0.920	0.25	0.97	<0.05
Breakfast consumption	-0.12	-0.230	0.84	0.71	0.89	<0.05
Convenience store use	0.15	1.110	3.03	0.72	12.72	0.13
High-carbohydrate meals	0.24	1.23	1.58	1.20	1.72	<0.05
Balanced meals	-0.17	-0.90	0.08	0.05	0.09	<0.05
Perceived dietary disruption	0.21	0.59	1.32	1.03	1.41	<0.05
Desire to diet	0.14	0.73	1.11	1.06	1.170	<0.05
Supplement intake	0.06	0.81	2.240	0.84	5.99	0.11

**Table 3:** Logistic Regression Analysis Predicting QIDS-J Score ≥ 6 Among Non-dormitory Students (n = 400) r, Correlation Coefficient; β, Standardized Partial Regression Coefficient; QIDS-J, Quick Inventory of Depressive Symptomatology-Japanese Version

Independent Variable	r	β	Odds Ratio	95% CI (Lower Bound)	95% CI (Upper Bound)	p-value
Sugary drinks	0.11	0.32	1.03	1.01	1.05	<0.05
Breakfast consumption	0.17	0.06	1.05	0.93	1.12	0.430
Convenience store use	-0.12	-0.18	0.74	0.74	1.01	0.24
High-carbohydrate meals	0.220	0.39	1.43	1.12	1.810	<0.05
Balanced meals	0.14	0.19	1.100	0.96	1.27	0.18
Perceived dietary disruption	0.19	0.17	1.130	0.95	1.35	0.17
Desire to diet	0.14	0.25	1.15	1.02	1.30	<0.05
Supplement intake	-0.19	-0.24	0.86	0.75	0.98	<0.05

The sub-analysis, which excluded dormitory students, extracted the consumption of high-carbohydrate meals and the use of supplements as factors related to QIDS-J scores.

Case study analysis

To deeply understand the complex interactions between dietary habits and depressive symptoms, which cannot be captured by quantitative data alone, a detailed examination was conducted on two individual cases classified as "very severe," based on their QIDS-J scores.

Case 1: A Vicious Cycle of Malnutrition and Depression Induced by an Inappropriate Desire to Diet

Case 1 was a 22-year-old female who exhibited very severe depressive symptoms with a QIDS-J score of 21. It is noteworthy that, despite having a low BMI of 16.9 (underweight), she possessed a strong "desire to diet," a factor shown to be associated with depressive symptoms in our main analysis.

Regarding her dietary habits, although she reported consuming nutritionally balanced meals daily, she almost always skipped breakfast, which, at first glance, may not seem like a major issue. However, she herself was aware that her eating habits had become irregular since entering university. The coexistence of this subjective awareness of an irregular diet, an objectively low body weight, and a strong desire to diet suggests an abnormal eating behavior stemming from a complex psychological state.

What is particularly remarkable about this case is that the participant was clearly aware of the link between her nutritional state and her mental health, and she accurately verbalized the vicious cycle (negative loop). Her written account indicates an awareness of a cycle where malnutrition leads to a decline in concentration and motivation, and the resulting lethargy makes the act of eating itself feel burdensome, leading to further malnutrition. This self-awareness is valuable qualitative data, demonstrating that a state of malnutrition can be a factor in maintaining and exacerbating depressive symptoms.

Case 2: Introspection on the "Reverse Causality" of Mental State Affecting Eating Behavior

Case 2 was a 24-year-old male who recorded the highest QIDS-J score (24) in this survey. Although he had a standard physique (BMI 22.8), he reported consuming high-carbohydrate meals - the factor presumed to have the strongest association with depression in the main analysis - almost every day, and not consuming any nutritionally balanced meals.

The importance of this case in the discussion lies in the participant's insightful introspection regarding the direction of causality between dietary habits and depressive symptoms. He self-analyzed a tendency to overeat when emotionally distressed, followed by self-loathing, which, in turn, led to more eating. Conversely, he noted that when his mental state was stable, he was able to enjoy balanced meals with others.

This description clearly illustrates, from the perspective of the individual, the possibility of "reverse causality" - a relationship that is difficult to determine in a cross-sectional study like this one - whereby depressive symptoms become a "cause" of unhealthy eating behaviors (such as high-carbohydrate diets). As the participant stated, although it is unclear whether a good mood dictates a good meal or vice versa, he suggests a strong association between the two, highlighting the significant impact of mental state on eating behavior.

## Discussion

This study investigated the association between dietary habits and depressive symptoms in 451 university students. The results showed a mean QIDS-J score of 5.8 for the entire sample and 6.0 for non-dormitory students, indicating a tendency toward mild depressive symptoms in the general student population. The fact that approximately 46.3% of the students had a QIDS-J score of 6 or higher, with the majority of them (191 individuals) being non-dormitory students, indicates that the mental health of university students is a significant issue - a trend consistent with previous studies [13-16].

The main analysis revealed that factors such as a high-carbohydrate diet, skipping breakfast, a nutritionally imbalanced diet, self-awareness of irregular eating habits, and a desire to diet were associated with the severity of depressive symptoms. In particular, a high-carbohydrate diet was suggested to have the largest impact. This is consistent with the findings of previous research showing that a high-monosaccharide diet can induce psychosis-like symptoms [17]. However, as this is a cross-sectional study, it is crucial to exercise caution in interpreting these associations as a unidirectional causal relationship, where diet leads to depression. Rather, the "reverse causality," where a depressive mood leads to irregular eating habits, is also highly plausible. This possibility is strongly supported by the introspection of the 24-year-old male in Case 2, who stated, "I tend to overeat when I'm emotionally distressed," and, "my mind might be affecting my diet." In other words, irregular eating habits are likely both a "cause" and a "consequence" of depressive symptoms, forming a vicious cycle of mutual influence. Furthermore, it is important to interpret the strength of these associations with caution, as they are based on self-reported data, which may be subject to recall bias.

On the other hand, the analysis of the entire student sample showed that the consumption of soft drinks "reduced" depressive symptoms, a result contrary to previous studies [18,19]. This is highly likely to have been influenced by selection bias and confounding from the inclusion of dormitory students (primarily student-athletes). It is strongly presumed that the lower QIDS-J scores among dormitory students are due to multiple protective factors against depressive symptoms, such as high levels of daily physical activity, close interpersonal relationships within their teams (social support), and a regular lifestyle. For them, the consumption of soft drinks is likely more related to energy replenishment after strenuous exercise and has a weak direct association with depressive symptoms. In fact, in the sub-analysis excluding this group of dormitory students, the consumption of soft drinks was associated with a worsening of depressive symptoms, which is consistent with previous research. This highlights the importance of considering lifestyle, particularly physical activity levels, when analyzing the association between depression and dietary habits.

Regarding other factors, skipping breakfast may lead to fluctuations in blood sugar levels during the day, contributing to mental instability, while a nutritionally imbalanced diet could exacerbate depressive tendencies by causing a deficiency in vitamins and minerals essential for brain function [20]. A desire to diet was also shown to be associated with depressive symptoms, and this statistical association can be concretely understood through the case of the 22-year-old female (Case 1). Despite her low BMI of 16.9, she had a strong desire to diet, and she was aware of a "negative loop," in which skipping breakfast and malnutrition led to a decline in concentration and motivation, further diminishing her energy to eat. This case vividly illustrates how an inappropriate desire to diet can damage mental health, not only through simple malnutrition but also through psychological stress and a decline in self-esteem.

From these results and the examination of individual cases, specific interventions for maintaining and improving the mental health of university students emerge. In cases like the female in Case 1, where malnutrition is linked to mental distress, the immediate priority is to restore physical and mental energy. As a means to this end, temporarily supplementing with amino acids (such as protein), which are precursors to serotonin involved in mood stability, and vitamins and minerals that aid in the synthesis of neurotransmitters, could be effective in breaking the negative loop [21]. On the other hand, in cases like the male in Case 2, where emotional turmoil leads to overeating, behavioral interventions are important in addition to a nutritional approach. As he himself stated, "when my mind is clear, I often eat with someone," creating an environment for eating balanced meals with others, instead of eating alone, could increase meal satisfaction and help suppress eating disorders caused by emotions [22,23].

Finally, the limitations of this study and future challenges should be addressed. As mentioned earlier, this is a cross-sectional study, so it is not possible to determine causal relationships. A significant methodological limitation is the use of an original, unvalidated dietary questionnaire. The lack of established reliability and validity for this instrument means that measurement error could have influenced the findings, and this should be considered when evaluating the results.

In addition, confounding factors that could affect depressive symptoms other than dietary habits, such as participation in part-time jobs or hobbies, stress levels, quality of social support, and the consumption of coffee or green tea - which have been reported to reduce the risk of depression - were not controlled for [24,25]. Furthermore, since the subjects were limited to students from a single university, the results of this study cannot be generalized to the entire university student population in Japan.

Based on these limitations, future research should aim to include more diverse student populations from multiple institutions to enhance generalizability. It is also crucial to control for a broader range of potential confounders, such as physical activity levels, specific caffeine intake, and detailed measures of social support. Moreover, the development and use of a validated dietary assessment tool specific to this population is highly recommended. Finally, longitudinal studies are essential to track changes over time and to more clearly elucidate the causal pathways between dietary habits and depressive symptoms.

## Conclusions

This study found that specific dietary tendencies may affect the severity of depressive symptoms, as measured by the QIDS-J. Unhealthy dietary habits, such as the consumption of soft drinks, skipping breakfast, high-carbohydrate diets, nutritionally imbalanced meals, irregular eating habits, and a desire to diet, may increase the severity of depressive symptoms. Among these, the consumption of high-carbohydrate meals was presumed to have the greatest impact on the severity of depression.

On the other hand, the effect of soft drink consumption differed between dormitory and non-dormitory students. In the dormitory group, higher consumption of soft drinks tended to be associated with lower severity of depressive symptoms, whereas, in the non-dormitory group, it was suggested that soft drink consumption might increase the severity of depressive symptoms. This is likely due to a bias in the dormitory group, which consisted of many male members of athletic clubs. While causality cannot be determined, it is conceivable that, for students engaged in strenuous sports, the consumption of soft drinks does not negatively affect depressive symptoms. This suggests the need for future studies to broaden the range of students and track changes over time.

## Appendices

**Table 4:** Questionnaire Details Including Measures of Dietary Habits and Open-Ended Question

Question Item	Options/Response Scale				
Q1 | What is your age?	years old				
Q2 | What is your gender?	1. Male	2. Female	3. Prefer not to say		
Q3 | What is your height in cm?	cm				
Q4 | What is your weight in kg?	kg				
Q5 | What is your current household composition?	1. Living alone	2. Living with parent(s) (nuclear family)	3. Living with grandparent(s) and parent(s) (three-generation household)	4. Living with friend(s)/acquaintance(s)	5. Living in a dormitory 6. Other
Q6 | How many 500 mL bottles of sugary drinks (e.g., juices, sports drinks, carbonated drinks, energy drinks containing artificial sweeteners or sugar) do you drink per week?	bottles				
Q7 | How many times do you eat breakfast per week?	times				
Q8 | How many days per week do you use a convenience store for meals?	days				
Q9 | Regarding falling asleep, please select the one option that applies to you:	1. No problem (or, Never takes more than 30 minutes to fall asleep)	2. Sometimes takes more than 30 minutes to fall asleep, but less than half the week	3. Takes more than 30 minutes to fall asleep, more than half the week	4. Takes more than 60 minutes to fall asleep, more than half the week	
Q10 | Regarding sleep during the night, please select the one option that applies to you:	1. No problem (Never wake up during the night)	2. Restless, light sleep with several brief awakenings	3. Wake up at least once each night, but go back to sleep easily	4. Wake up more than once each night and stay awake for 20 minutes or more, more than half the week	
Q11 | Regarding waking up too early, please select the one option that applies to you:	1. No problem (or, Mostly wake up no more than 30 minutes before I need to get up)	2. Wake up more than 30 minutes before I need to get up, more than half the week	3. Almost always wake up more than 1 hour before I need to, but eventually fall back asleep	4. Wake up more than 1 hour before I need to and cannot get back to sleep	
Q12 | Regarding sleeping too much, please select the one option that applies to you:	1. No problem (Do not sleep too much at night and do not nap during the day)	2. Sleep about 10 hours in a 24-hour period, including naps	3. Sleep about 12 hours in a 24-hour period, including naps	4. Sleep more than 12 hours in a 24-hour period, including naps	
Q13 | Regarding feeling sad, please select the one option that applies to you:	1. I do not feel sad	2. I feel sad less than half the time	3. I feel sad more than half the time	4. I feel sad nearly all the time	
Q14 | Regarding decreased appetite, please select the one option that applies to you:	1. My appetite is unchanged from usual, or increased	2. I eat somewhat less often or lesser amounts than usual	3. I eat much less than usual and have to push myself to eat	4. I eat hardly anything all day (24 hours) and only eat if strongly urged by others or myself	
Q15 | Regarding increased appetite, please select the one option that applies to you:	1. My appetite is unchanged from usual, or decreased	2. I feel the need to eat more frequently than usual	3. I regularly eat more often and/or larger amounts than usual	4. I feel driven to overeat at meal times and between meals	
Q16 | Regarding weight loss (in the last 2 weeks), please select the one option that applies to you:	1. My weight has not changed, or it has increased	2. I feel I have lost a little weight	3. I have lost more than 1 kg	4. I have lost more than 2 kg	
Q17 | Regarding weight gain (in the last 2 weeks), please select the one option that applies to you:	1. My weight has not changed, or it has decreased	2. I feel I have gained a little weight	3. I have gained more than 1 kg	4. I have gained more than 2 kg	
Q18 | Regarding concentration/decision making, please select the one option that applies to you:	1. My concentration and decision-making ability are unchanged from usual	2. I occasionally feel indecisive or find my attention wandering	3. Most of the time, I struggle to focus my attention or make decisions	4. I cannot concentrate enough to read or make even minor decisions	
Q19 | Regarding your view of yourself, please select the one option that applies to you:	1. I see myself as equally worthwhile and deserving as other people	2. I am more self-blaming than usual	3. I largely believe that I cause problems for others	4. I think almost constantly about my major and minor defects	
Q20 | Regarding thoughts of death or suicide, please select one option:	1. I do not think of death or suicide	2. I feel that life is empty or wonder if it is worth living	3. I think of suicide or death several times a week for several minutes	4. I think of suicide or death several times a day in detail, or I have made specific plans for suicide or have actually tried to take my life	
Q21 | Regarding general interest, please select one option:	1. My interest in other people or activities is unchanged from usual	2. I notice that I am less interested in people or activities than usual	3. I find I have interest in only one or two of my formerly pursued activities	4. I have virtually no interest in formerly pursued activities	
Q22 | Regarding energy level, please select one option:	1. My energy level is unchanged from usual	2. I get tired more easily than usual	3. I have to make a great effort to start or finish my usual daily activities (e.g., shopping, homework, cooking, going to work)	4. I am unable to carry out most of my usual daily activities because I just do not have the energy	
Q23 | Regarding feeling slowed down, please select the one option that applies to you:	1. I think, speak, and move at my usual rate of speed monotonous or flat	2. I find that my thinking is slowed down or my voice sounds	3. It takes me several seconds to respond to most questions and I am sure my thinking is slowed	4. I am often unable to respond to questions without extreme effort	
Q24 | Regarding feeling restless, please select the one option that applies to you:	1. I do not feel restless	2. I am often fidgety, wringing my hands, or need to shift how I am sitting	3. I have impulses to move about and am quite restless	4. At times, I am unable to stay seated and need to walk around	
Q25 | Do you sometimes have meals consisting only of high-carbohydrate items like noodles (ramen, pasta, soba, udon, etc.), rice bowls (fried rice, curry, beef bowls, etc.), or sweet buns?	1. Almost every day	2. More than half the week	3. About half the week	4. Less than half the week	5. Never
Q26 | Do you eat nutritionally balanced meals considering staple food (rice, bread, noodles), main dish (meat, fish, eggs, soy products), side dish (vegetables, mushrooms, potatoes, seaweed), dairy products, fruits, etc.?	1. Almost every day	2. More than half the week	3. About half the week	4. Less than half the week	5. Never
Q27 | Compared to before you became a university student, do you feel your current eating habits are more disrupted?	1. Agree	2. Somewhat agree	3. Somewhat disagree	4. Disagree	
Q28 | Do you want to lose weight from your current weight by dieting?	1. Agree	2. Somewhat agree	3. Somewhat disagree	4. Disagree	
Q29 | Do you use supplements or health補助食品 (health auxiliary foods/fortified foods)?	1. Almost every day	2. More than half the week	3. About half the week	4. Less than half the week	5. Never
Q30 | (Question for those who answered 1-4 on Q29) Please select all supplements, etc., that you use (multiple answers allowed):	1. Protein (incl. protein bars, etc.) or Amino Acids	2. Vitamin B	3. Vitamin C	4. Iro	5. Intestinal remedies (probiotics/prebiotics)
	6. Zinc	7. Calcium	8. Magnesium	9. Omega-3 fatty acids	10. FOSHU (Food for Specified Health Uses) products
	11. Solid nutritional supplements (e.g., CalorieMate)	12. Jelly-type nutritional supplements (e.g., Weider in Jelly)	13. Complete nutrition bread	14. Other	
Q31 | Please write your opinion (within 100 characters) on whether you think nutrition is related or unrelated to mental health.	Free description				
